# Anecdote

**Published:** 1855-07

**Authors:** 


### ANECDOTE.

We copy the following anecdote which, although not new, is better told than we have ever before seen it, by our esteemed cotemporary the Virginia Medical and Surgical Journal. It describes a class of physicians, good specimens of which may be found much this side of France. But to the story; it is entitled—

The Interrogative Doctor.—A poor devil was once carried into a French hospital, run through the body with a roasting-spit. The surgeon on duty approached, gazed at the patient with a mediative air, and, after a time, addressed him as follows: What is your name? Your age? Your profession? What disease have you had in the course of your life? Are you well fed and lodged? Is your sleeping apartment well ventilated? What relations have you? Are they subject to any disease? Of what did they die? Have you children? Is their health good? How were you injnred? To each of these questions the poor wounded man replied painfnlly, gasping, after each answer: The spit! Take away the spit! . . . . Have patience, my friend; do you suffer much pain? What sort of pain do you suffer? Oh! doctor, the spit, the spit! Was the blood you lost red or black? Do you breathe easily? The spit! Have you any cough? Do you raise anything? Let me see your tongue. Have you a bad taste in your mouth? Have you a good appetite? Are your bowels in order? Have you passed any worms recently? Can you lie on one side as well as the other? Is your water high colored? Has this accident happened to you often before? Are your family subject to it? The spit, the spit! One moment; let me feel your pulse. And the doctor, with a magisterial air, examined all the functions, percussed, succussed, auscultated, and the patient expired, just as he was preparing to remove the instrument of impalement.

---

We laid aside the following anecdote of the Rev. Sidney Smith for a previous number, but in some way it was overlooked by the compositor. It is too good, however, to keep:

A Decided Sell.—Lady Cubebs had a great passion for the garden and the hot-house, and when she got hold of a celebrity like the Reverend Sidney, was sure to dilate upon her favorite subject. Her geraniums, her Auriculas, her Dahlias, her Carnations, her Acacias, her Lillia Regia, her Ranunculus, her Marygolds, her Peonies, her Rhododendron Procubens, Mossy Pompone and Rose pubescens, were discussed with all the flow of hot-house rhetoric. "My Lady," asked the Reverend wit, "did you have a Psoriasis Septennis?" "Oh yes—a most b-e-a-u-tiful one. *I gave it to the Archbishop of Canterbury.* Dear man!—and it came out *so* in the spring!"

